# Outcomes of patient education in adult oncologic patients receiving oral anticancer agents: a systematic review protocol

**DOI:** 10.1186/s13643-023-02229-x

**Published:** 2023-04-20

**Authors:** Federico Fonda, Sonja Cedrone, Ivana Sartor, Lucia Cadorin

**Affiliations:** 1 Department of Anaesthesia and Intensive Care, Azienda Sanitaria Universitaria Friuli Centrale, University Hospital of Udine, Udine, 33100 Italy; 2 Centro di Riferimento Oncologico di Aviano (CRO) IRCCS, Aviano, 33081 Italy

**Keywords:** Patient education, Outcomes, Oral anticancer agents, Cancer, Systematic review protocol

## Abstract

**Background:**

A large variety of oral anticancer agents have become available and while at first glance these therapies appear to provide only benefits, patients have expressed their need for educational interventions and raised safety issues. Although both patients and providers have recognized patient education’s importance, and an interplay with safety has been acknowledged, no systematic reviews of the literature that summarize all of the current evidence related to patient education’s outcomes for patients who receive oral anticancer agents have been performed to date. Accordingly, this systematic review will attempt to fill the gap in the literature as well as to map (1) contents, (2) methodologies, (3) settings, (4) timing/duration, and (5) healthcare professionals involved.

**Methods:**

This protocol is being reported in accordance with the Preferred Reporting Items for Systematic Reviews and Meta-Analyses guidelines. A systematic review will be performed. Studies that targeted eligible adult patients (≥ 18 years old) in hospital, outpatient, and home settings, and reported patient education’s outcomes for those taking oral anticancer agents will be included. Searches will be conducted in PubMed/MEDLINE, CINAHL, Embase, and Scopus, and gray literature will be also sought. Two researchers will screen the search results independently and blindly in two phases: (1) title/abstract screening and (2) full-text screening using the Rayyan AI platform. An electronic data extraction form will be implemented and piloted, and then, two trained data extractors will extract the data cooperatively. Thereafter, a quality appraisal will be conducted using the Critical Appraisal Tools from The Joanna Briggs Institute. The results will be analyzed, grouped, clustered into categories, and discussed until a consensus is reached. Emerging evidence will be synthesized narratively and reported in accordance with the synthesis without meta-analysis guidelines.

**Discussion:**

The systematic review’s results will be relevant to (1) policymakers and management at an institutional level, and (2) for clinical practice, in an evidence-based paradigm, potentially leading to a quality improvement with respect to safety and patient satisfaction.

**Systematic review registration:**

PROSPERO CRD42022341797

**Supplementary Information:**

The online version contains supplementary material available at 10.1186/s13643-023-02229-x.

## Background

During the past several years, a large variety of antineoplastic agents for oral administration has become available [1, 2] and led to a shift in cancer treatment from the hospital to the home setting [3]. In a survey of patients’ preferences for oral anticancer agents (OAA) [4], the majority (89.32%, *n* = 90) declared that they preferred oral to intravenous chemotherapy. The primary reasons given were convenience, concerns about intravenous access, and perceived environmental control [4]. It has been reported also that patients may have misconceptions about OAA with respect to their side effects and efficacy [5]. In this evolving scenario, special care must be given to oncologic patients who often undergo a multi-faceted and multi-professional treatment pathway that requires them to have timely and accurate information related to their needs. While at first glance, OAA therapies appear to provide only benefits [5], a qualitative study found that patients expressed their need for educational interventions, raised safety issues related to OAA, and were concerned about both identifying and managing their side effects [6]. An interplay between safety and patient education (PE) has been raised and is acknowledged that OAA are prescribed best in combination with structured educational efforts [5]. PE, which is defined as “a process of assisting consumers of health care to learn how to incorporate health related behaviors into everyday life with the purpose of achieving the goal of optimal health” [7, 8], has long been recognized as “an essential component of effective healthcare delivery” [9] in which registered nurses (RNs) are seen as “patient teachers” [10] with the support of patients who are recognized as “equal partners” [11]. Hence, oncology RNs are required to find effective ways to educate patients about cancer diagnosis, treatment, and symptom management [12].

### Objectives

Both patients and providers have reported the importance of PE for those taking OAA [6, 13]. O’Neill and colleagues also highlighted PE’s crucial role, particularly when OAA are taken outside of the hospital in a community setting [13]. Although PE has been documented as an important area of intervention that enhances adherence to OAA [14, 15], to the best of our knowledge, there are no systematic reviews (SRs) of the literature to date that have summarized all of the current evidence about the outcomes of PE for patients receiving OAA. To fill this gap in the literature, the underlying research question formulated following the Population (P), Intervention (I), Comparison (C), and Outcomes (O) framework [16, 17] is: what are the documented outcomes (O) of patient education interventions (I) for adult patients with solid/oncohematological cancer who receive OAA (P) compared to no structured PE interventions (C)?

The secondary objectives will be to describe systematically the (1) content, (2) methodologies, (3) setting, (4) timing/duration, and (5) healthcare professionals involved in documented PE interventions that target patients who take OAA.

## Methods

### Research design and methodology

We will perform a systematic review of the literature (SR) that will be guided by the standards of reporting of the Preferred Reporting Items for Systematic Reviews and Meta-Analyses (PRISMA) 2020 [18]. The SR protocol is being reported in accordance with the Preferred Reporting Items for systematic reviews and Meta-Analyses for Systematic Review Protocols (PRISMA-P) 2015 [19]. The SR was also registered prospectively in the International Prospective Register of Systematic Reviews (PROSPERO) (Registration number CRD42022341797). The complete PRISMA-P Checklist is available as Additional file 1.

### Protocol development

To define the SR protocol and perform the subsequent SR, a purpose-built research team was constituted and multiple stakeholders from an Italian National Cancer Institute were involved. A total of seven RNs, two physicians, and two pharmacists were consulted, including a clinical trial nurse with extensive clinical experience with patients who are receiving OAA and PE interventions (IS), a pharmacist with experience in PE programs’ management, and a pharmacist from the pharmacy’s clinical desk dedicated to the OAA’s distribution, counseling, and pharmacovigilance. A senior PhD researcher (LC) with experience in both quantitative/qualitative studies and SRs in the field of educational research was appointed supervisor of the research team.

**Fig. 1:**
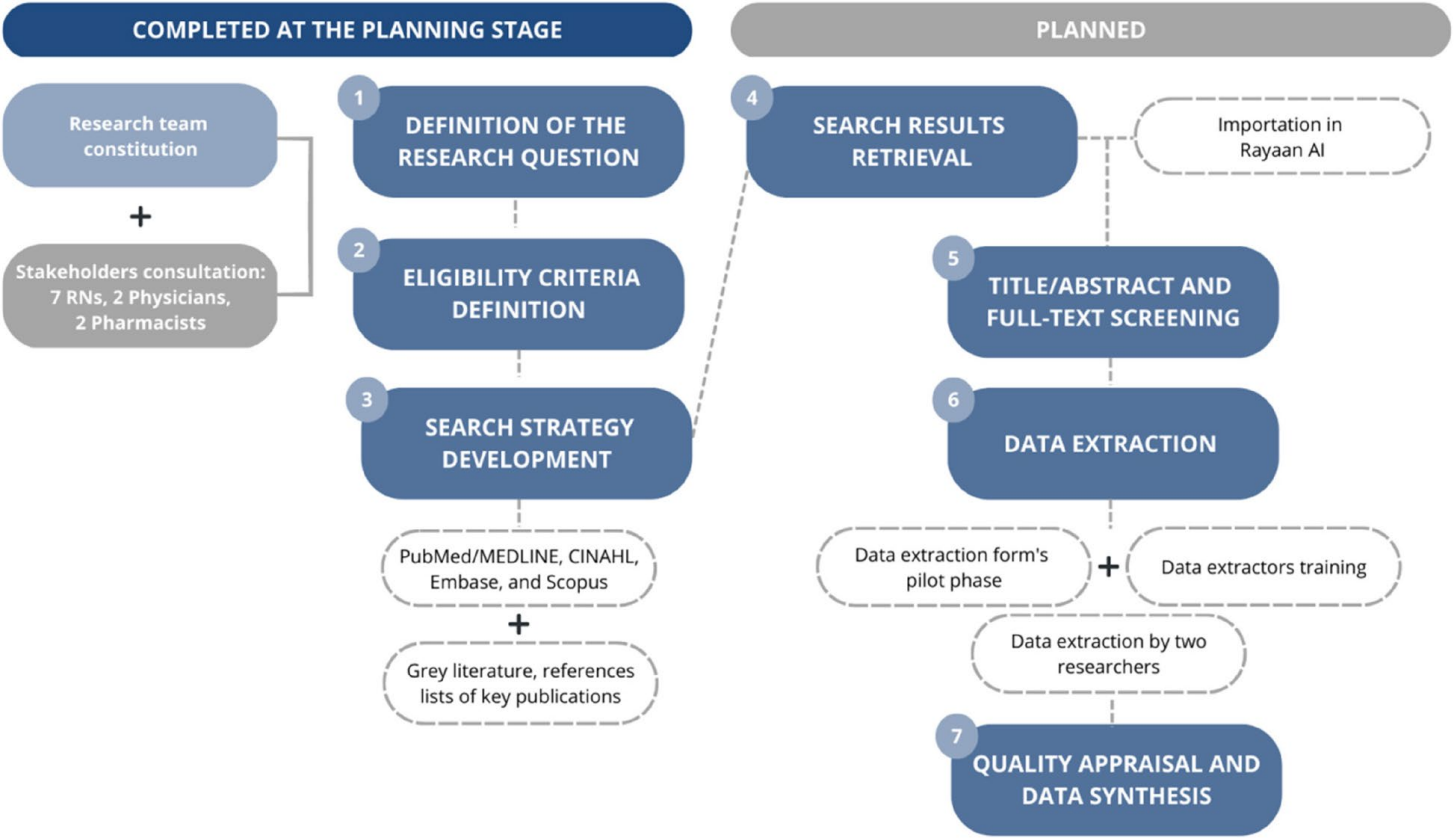
Systematic review’s protocol development phases. Legend. RNs, registered nurses; MEDLINE, Medical Literature Analysis and Retrieval System Online; CINAHL, Cumulative Index to Nursing and Allied Health Literature; Embase, Excerpta Medica database

As shown in Fig. 1, the research team first developed the research question and established the SR’s primary and secondary objectives in detail [20] using the PICO framework [16, 17] and then defined the eligibility criteria and search strategy accordingly. The processes of retrieving the search results, screening the title/abstract and full-text, extracting and synthesizing the data, and appraising their quality have also been planned and are described in detail in the SR protocol.

### Eligibility criteria

#### Study types, time restrictions, and language

Randomized controlled trials (RCTs), clinical trials (CT), prospective and retrospective cohort studies, case-control studies, and cross-sectional studies will be included, while qualitative studies, editorials, letters, reports, commentaries, books, dissertations, conference papers, and proceedings will be excluded. No time restrictions will be applied to summarize the entire existing literature and provide a comprehensive review [21]. Only studies written in English will be included.

#### Population and setting

Adult patients (≥ 18 years old) of both genders from any country in hospital, outpatient, and home settings who are diagnosed with solid or oncohematological cancer and prescribed OAA will be included, while studies of pediatric populations (< 18 years old) will be excluded.

#### Intervention and comparison

All types of PE interventions that provide contents related to OAA, including, but not limited to, basic information about the drug, the therapeutic schedule, managing collateral effects, administration methods, reporting adverse events, and monitoring will constitute the intervention group, and will be compared to those with no structured PE intervention who receive only standard information.

#### Outcomes

The research team (FF, IS, LC) and the stakeholders held multiple meetings to define the expected outcomes at this stage. It is hypothesized that PE outcomes may include the following: number of reports of adverse events, toxicity related to OAA, hospital/emergency care admissions, the number of nurse/physician/pharmacist consults, adherence to follow-ups, quality of life (QoL), use of alternative pharmacological and non-pharmacological therapeutic approaches, caregiver burden, and adherence to the medication prescribed. Other outcomes that may emerge during the literature analysis will be considered as well.

A complete overview of the eligibility criteria and their rationale is provided in Table 1.

**Table 1:** Eligibility criteria and rationale

**Inclusion criteria**	**Exclusion criteria**	**Rationale**
Randomized controlled trials (RCT), clinical trials (CT), prospective and retrospective cohort studies, case-control-studies, and cross-sectional studies	Qualitative studies, editorials, letters, reports, commentaries, books, dissertations, conference papers, and conference proceedings	/
No time restrictions	/	To summarize the entire existing literature and provide a comprehensive review [21]
Articles written in English language	Articles written in languages other than English	To seek evidence that targets an international audience
Patients (≥ 18 years old) of any genders diagnosed with solid or oncohematological cancer who are prescribed OAA	Pediatric population (< 18 years old) or patients without diagnosis of oncohematological cancer	A difference is expected in approaches and interventions that target the pediatric population. For instance, PE interventions that target OAA may be directed to parents, and thus are very different in contents and methodology.
Hospital, outpatient, and home setting	/	All of the settings will be explored
All types of PE interventions	/	No strict exclusion criteria of PE interventions have been defined, as the SR’s secondary objective will be to describe systematically the documented PE interventions that target OAA in their content, methodologies, setting, timing/duration, and healthcare professionals involved
Comparison with usual care which includes standard information	/	/
Studies reporting PE outcomes	Studies that do not report PE outcomes will be excluded	Studies that do not report PE outcomes will be excluded

*RCT* Randomized controlled trials, *CT* Clinical trials, *PE* Patient education, *OAA* Oral anticancer agents, *SR* Systematic review

#### Information sources

A systematic search of the literature available will be conducted in the following electronic databases: PubMed/Medical Literature Analysis and Retrieval System Online (MEDLINE), Cumulative Index to Nursing and Allied Health Literature (CINAHL), Excerpta Medica atabase (Embase), and Scopus. In addition, gray literature, such as non-indexed journals and professional associations’ websites will be searched. The reference lists of key publications will be taken into consideration and two researchers (FF, LC) will screen them in-depth throughout to identify additional relevant studies not retrieved in the online searches [16].

#### Search strategy

After consultations among the team members (FF, IS, LC) and stakeholders, relevant keywords and medical subject headings (MeSH) terms were identified. Each keyword/MeSH term was combined using the Boolean operators AND/OR/NOT. LC and a senior librarian who was involved in both the conceptualization and preparation of the search strategy supervised the process. The complete search strategy for each database is described in Additional file 2.

#### Screening

The search results will be screened in two phases: (1) title/abstract screening and (3) full-text screening. This part of the SR will attempt to include only relevant studies and exclude irrelevant articles [22]. FF will import the search results into the Rayyan AI platform [23], and after duplicate results have been removed, title/abstract screening will be conducted. Subsequently, full-text screening of studies that meet the inclusion criteria will be performed. Two researchers (FF, LC) will conduct the screening independently and blindly, and a Cohen’s kappa score of >0.6 will be considered acceptable [24]. In case of disagreements about article eligibility, a third researcher will be consulted (IS), and the final decision will be made during discussion until consensus is reached [16].

#### Data extraction

An electronic data extraction form will be implemented [25] and piloted with at least two of the articles selected to ensure its usefulness, appropriateness, and feasibility [16, 26]. The entire team will discuss the results of the pilot and the data extraction form will be adjusted accordingly. Two data extractors (SC, IS) with knowledge of PE, OAA, and research methods will extract the data cooperatively [25]. In addition, the data extractors will be provided with training to ensure that they are familiar with the tool and to enhance the results’ trustworthiness [26]. A third researcher will be involved (FF) to resolve disagreements in the data extraction, and the final decision will be made upon discussion until consensus is reached [16, 25].

The following data will be extracted:

1. Author(s), year of publication, and country.
2. Study design and objectives.
3. Setting and population characteristics (including oncologic disease and OAA drug prescribed).
4. Content(s) of the PE intervention.
5. PE intervention methodologies adopted/studied (i.e., written, verbal, etc.).
6. Timing and duration of the PE intervention.
7. Healthcare professionals involved in the PE process.
8. Comparison.
9. Outcomes explored with measurement tools/measurement of effects.

#### Quality appraisal

Two researchers (FF, LC) will appraise the quality of the studies included independently using the Critical Appraisal Tools from The Joanna Briggs Institute [27], which provides thirteen checklists that contain from a minimum of six to thirteen items, respectively. For each item, “yes,” “no,” “unclear,” or “not applicable” answers are available. No studies will be excluded during the quality appraisal phase. The quality assessment will be considered an advantage to understand the study results’ meaning and weight better. A Cohen’s kappa score of >0.6 will be considered acceptable in quality appraisal as well [24].

#### Data synthesis

The completed electronic data extraction form will be shared among all of the research team members. Initially, the data will be aggregated by study design, setting, and main PE outcomes. Patterns and relations in the results will be discussed in multiple meetings until consensus is reached. The results will be analyzed, grouped, and assigned to categories according to the studies’ similarities and differences, with particular emphasis on outcomes and (1) content, (2) methodologies, (3) setting, (4) timing/duration, and (5) healthcare professionals involved. Because of the considerable heterogeneity expected given the broad eligibility criteria in the study design, population, setting, PE interventions, and outcomes, the emerging evidence will be synthesized narratively [28], and the results will be reported according to the Synthesis without meta-analysis (SWiM) guidelines [29]. A textual description of the findings, as well as tables, the vote counting technique, and figures will be adopted when appropriate [28].

## Discussion

### Relevance of the systematic review

The SR will address the PE outcomes documented for adult oncologic patients who are receiving OAA, as well as describe the PE interventions’ contents, methodologies, setting, timing/duration, and the healthcare professionals involved. Therefore, the results will be relevant to policymakers and management, as they will inform policies and provide guidance in structuring PE programs for OAA patients at an institutional level, and for clinical practice in an evidence-based paradigm , they will advance the knowledge and awareness of PE outcomes for patients receiving OAA, and potentially lead to a change in the practice that ensures quality improvement with respect to safety and patient satisfaction [5, 6, 12].

### Limitations and strategies

The SR has certain limitations. Only articles written in English will be included, as it is intended to seek evidence that targets an international audience, which could potentially lead to selection bias. No individual search within the most relevant journals in the field is planned, although they are expected to be indexed in the broad databases that will be searched. Finally, negative or neutral results of PE interventions may have not been published, which could introduce an involuntary publication bias in the SR.

As a strategy to improve the SR’s transparency, all important amendments to the protocol in the final SR will be reported and described in-depth in an appendix that provides the rationale and a full description of the change(s).

## Supplementary Information

**Additional file 1.** “PRISMA- P 2015 Checklist”. Microsoft Word document.

**Additional file 2.** “Search strategy”. Microsoft Word document.

## Data Availability

All data generated or analyzed during this study will be available in another published article.

## Abbreviations

OAA: Oral anticancer agents
PE: Patient education
RN(s): Registered nurse(s)
SR(s): Systematic review(s)
PRISMA: Preferred Reporting Items for Systematic Reviews and Meta-Analyses
PRISMA-P: Preferred Reporting Items for Systematic Reviews and Meta-Analyses for Systematic Reviews Protocols
PROSPERO: International Prospective Register of Systematic Reviews
RCT: Randomized controlled trial(s)
CT: Clinical trial(s)
QoL: Quality of life
MEDLINE: Medical Literature Analysis and Retrieval System Online
CINAHL: Cumulative Index to Nursing and Allied Health Literature
Embase: Excerpta Medica dataBASE
MeSH: Medical Subject Headings
SWiM: Synthesis without meta-analysis guidelines
