# Supplementary material for: Outcomes of patient education in adult oncologic patients receiving oral anticancer agents: a systematic review protocol
Source: Syst Rev. 2023 Apr 20;12:69. doi: 10.1186/s13643-023-02229-x (PMC10120216; doi:10.1186/s13643-023-02229-x)
Supplement: Supplementary file 2 — Additional file 2. “Search strategy”. Microsoft Word document. [file 13643_2023_2229_MOESM2_ESM.docx]

**Additional file 2. Search strategy**

| **Database** | **PubMed/Medical Literature Analysis and Retrieval System Online (MEDLINE)** |
| --- | --- |
| Search query | (("Patient Education as Topic"[Mesh] OR "Counseling"[Mesh] OR COUNSELING[TIAB] OR ((Self Care[Mesh] OR Self-Management[Mesh]) AND education) OR "patient education"[All Fields] OR "Patient Education Handout" [Publication Type] OR Health Education[MH:NOEXP] OR Consumer Health Information[MH] OR "patients education"[all fields] OR (patient*[tiab] AND education*[tiab]) OR "therapeutic education"[All Fields] OR "patient training"[All Fields] OR "patients training"[all fields] OR "patient information"[All Fields] OR patients information[All Fields]) AND (THERAP*[TIAB] OR DRUG THERAPY OR therapeutic OR "Antineoplastic Agents" [Pharmacological Action] OR NEOPLASMS/DRUG THERAPY[MH] OR chemotherap*[tiab] OR treat*[tiab] OR "Antineoplastic Agents"[Mesh] OR "Consolidation Chemotherapy"[Mesh] OR "Induction Chemotherapy"[Mesh] OR "Maintenance Chemotherapy"[Mesh] OR "Chemotherapy, Adjuvant"[Mesh] OR "ANTICANCER THERAPY"[TIAB] OR "ANTINEOPLASTIC THERAPY"[TIAB] ) AND (ORAL[TIab] OR "Administration, Oral"[Mesh] OR "ORAL ANTICANCER"[all fields] OR ORAL ONCOLYTIC OR "oral drug therapy"[All Fields] OR "oral treated"[All Fields] OR "oral treatment"[All Fields] OR "oral treatments"[All Fields] OR "oral therapy"[All Fields] OR "oral chemotherapy"[All Fields] OR "ORAL ANTINEOPLASTIC"[All Fields] OR "oral administration"[All Fields] OR "administration oral"[All Fields] OR "anticancer oral"[All Fields] OR "oral cancer therapy"[All Fields] OR "oral cancer treated patients"[All Fields] OR "oral cancer treatment"[All Fields] OR "oral cancer drug"[All Fields] OR "oral cancer drugs"[All Fields] OR "oral cancer therapeutics"[All Fields] OR "oral cancer therapies"[All Fields] OR "oral agent"[All Fields] OR "oral agents"[All Fields]) AND (neoplasms[MH] OR cancer[TIAB] OR ONCOLOGY[tiab] OR oncolog*[tiab] OR oncohematolog*[tiab] OR leukemia OR lymphoma*[tiab] OR myeloma*[tiab]) AND (ita[la] OR eng[la])) NOT (review[pt] OR systematic review[pt] OR oral care[ti] OR oral cancer[ti] OR oral mucositis[ti] OR oral health[ti] OR dent*[ti] OR odont*[ti] OR oral cavity[ti] OR "oral hygiene"[ti] OR hpv[ti] OR papilloma*[ti] OR oral feeding[ti] OR oral nutrition[ti] OR oral squamous[ti] OR oral mucosal[ti]) |
| **Database** | **Cumulative Index to Nursing and Allied Health Literature (CINAHL)** |
| Search query | ((MH "Neoplasms+") OR (MH "Leukemia+") OR (MH "Lymphoma+") OR (MH "Hodgkin's Disease") OR TI (leukem* OR lymphom* OR cancer OR oncolog* OR tumor* OR tumour* OR myeloma*) OR AB (leukem* OR lymphom* OR cancer OR oncolog* OR tumor* OR tumour* OR myeloma*) ) AND ( (MH "Hyperthermic Intraperitoneal Chemotherapy") OR (MH "Induction Chemotherapy") OR (MH "Consolidation Chemotherapy") OR (MH "Chemotherapy, Adjuvant+") OR (MH "Chemotherapy, Cancer+") OR (MH "Chemotherapy Care (Saba CCC)") OR (MH "Chemotherapy Management (Iowa NIC)") OR (MH "Antineoplastic Agents, Combined") OR "CHEMOTHERAP*" OR (MH "Drug Therapy+") OR (MH "Neoplasms+/DT") OR TI treat* OR TI therap* OR chemotherap*) AND ((MH "Administration, Oral+") OR (MH "Medication Administration: Oral (Iowa NIC)") OR TI oral OR AB oraL ) AND ( "therapeutic education" OR "patient* education" OR TI(patient* AND education) OR AB (patient* AND education) OR "therapeutic education" OR "patient* training" OR "patient* information" OR (MH "Patient Education (Iowa NIC)+") OR (MH "Health Education") OR ((MH "Self Care+" OR MH "Self-Care Component (Saba CCC)+" OR MH "Self-Care Assistance: Dressing-Grooming (Iowa NIC)" OR MH "Self-Care Assistance: Bathing-Hygiene (Iowa NIC)" OR MH "Self Care Agency" OR MH "Feeding Self Care Deficit (NANDA)" OR MH "Dressing-Grooming Self Care Deficit (NANDA)" OR MH "Self-Care: Parenteral Medication (Iowa NOC)" OR MH "Self-Care: Instrumental Activities of Daily Living (Iowa NOC)") AND education) OR (MH "Health Information+") )) NOT PT(abstract OR proceedings OR review OR doctoral dissertation OR ceu) |
| **Database** | **Excerpta Medica dataBASE (Embase)** |
| Search query | #6 AND ([adult]/lim OR [aged]/lim OR [middle aged]/lim OR [very elderly]/lim OR [young adult]/lim) #6 #3 AND #4 AND #5 #5 'oral drug administration'/exp OR oral:ti #4 'cancer therapy'/exp OR 'antineoplastic agent'/exp OR chemotherap*:ti,ab #3 'patient education'/exp OR 'patient education material'/exp OR 'health education'/de OR 'patient counseling'/exp OR 'patient information'/exp OR 'patient information leaflet'/exp OR (patient*:ti,ab AND education*:ti,ab) OR 'therapeutic education':ti,ab #2'patient counseling'/exp #1'patient education' |
| **Database** | **Scopus** |
| Search query | TITLE-ABS-KEY ( patient* ) AND ( TITLE ( education* OR counseling OR "Self Care" OR "Self Management" OR information ) OR KEY ( education* OR counseling OR "Self Care" OR "Self Management" OR information ) ) AND TITLE-ABS-KEY ( oral ) W/3 TITLE-ABS-KEY ( administration OR therapy OR medication OR therapeutic OR chemotherap* OR treatment OR anticancer OR anti-cancer ) AND TITLE-ABS-KEY ( neoplasm* OR cancer OR oncolog* OR oncohematolog* OR leukemia OR lymphoma* OR myeloma* ) AND NOT TITLE ( mucositis OR mouth OR stomatit* OR dent* OR odont* OR "oral health" OR periodont* OR "oral cancer" ) AND ( LIMIT-TO ( DOCTYPE , "ar" ) OR LIMIT-TO (DOCTYPE , "sh" ) OR LIMIT-TO ( DOCTYPE , "ed" ) OR LIMIT-TO ( DOCTYPE , "le" ) OR LIMIT-TO ( DOCTYPE , "Undefined" )) |
